# Metagenomic-based Surveillance of Pacific Coast tick *Dermacentor occidentalis* Identifies Two Novel Bunyaviruses and an Emerging Human Ricksettsial Pathogen

**DOI:** 10.1038/s41598-017-12047-6

**Published:** 2017-09-25

**Authors:** Jerome Bouquet, Michael Melgar, Andrea Swei, Eric Delwart, Robert S. Lane, Charles Y. Chiu

**Affiliations:** 1 Department of Laboratory Medicine, University of California, San Francisco, CA 94143 USA; 2 Department of Environmental Science, Policy and Management, University of California, Berkeley, CA 94720 USA; 3 Department of Biology, San Francisco State University, San Francisco, CA 94132 USA; 4 Blood Systems Research Institute, San Francisco, CA 94118 USA

## Abstract

An increasing number of emerging tick-borne diseases has been reported in the United States since the 1970s. Using metagenomic next generation sequencing, we detected nucleic acid sequences from 2 novel viruses in the family Bunyaviridae and an emerging human rickettsial pathogen, *Rickettsia philipii*, in a population of the Pacific Coast tick, *Dermacentor occidentalis* in Mendocino County sampled annually from 2011 to 2014. A total of 250 adults of this human-biting, generalist tick were collected from contiguous chaparral and grassland habitats, and RNA from each individually extracted tick was deep sequenced to an average depth of 7.3 million reads. We detected a *Francisella endosymbiont* in 174 ticks (70%), and *Rickettsia spp*. in 19 ticks (8%); Rickettsia-infected ticks contained *R. rhipicephali* (16 of 250, 6.4%) or *R. philipii* (3 of 250,1.2%), the agent of eschar-associated febrile illness in humans. The genomes of 2 novel bunyaviruses (>99% complete) in the genera *Nairovirus* and *Phlebovirus* were also identified and found to be present in 20–91% of ticks, depending on the year of collection. The high prevalence of these bunyaviruses in sampled *Dermacentor* ticks suggests that they may be viral endosymbionts, although further studies are needed to determine whether they are infectious for vertebrate hosts, especially humans, and their potential role in tick ecology.

## Introduction

*Dermacentor* is a genus of hard-bodied ticks in the family *Ixodidae* that utilizes small and large mammals as primary hosts. Certain species occasionally attach to humans and serve as vectors of microbial disease agents. The Pacific Coast tick (*D. occidentalis*) is naturally infected with the human pathogens *Anaplasma phagocytophilum, Borrelia burgdorferi* and *Ehrlichia chaffeensis*, as well as the veterinary pathogens *A. bovis* and *A. ovis* ^1^. *D. occidentalis* is also though to be the primary vector for *R. philipii* (previously known as the unclassified *Rickettsia* 364D), a recently recognized cause of eschar-associated illness in California^2^.

Unlike PCR amplicon sequencing, which is limited to a few predefined targets, unbiased metagenomic next-generation sequencing (mNGS), otherwise known as deep sequencing, can be used to identify novel and emerging human pathogens circulating in the tick vector. For example, Heartland virus, discovered in Missouri in 2012 using mNGS and transmitted by the lone star tick (*Amblyomma americanum*), is a potential cause of febrile illness and death in humans^3^ and has since been detected in mammalian hosts from 13 U.S. states^4^. Deep sequencing was also recently used to identify Bourbon virus, a novel virus in the genus *Thogotovirus* associated with a fatal case of febrile tick-borne illness in Kansas in 2015^5^.

Most prior metagenomic studies have sequenced pools of ticks rather than testing ticks individually, sampled ticks at single time points rather than longitudinally over multiple years, and have focused on either the virome or bacterial microbiome but not both. The SURPI (“sequence-based ultra-rapid pathogen identification”) pipeline uses the entirety of the National Center for Biotechnology Information (NCBI) GenBank database to identify all microbial agents – viruses, bacteria, fungi, and parasites – in mNGS sequencing data on the basis of sequence homology^6^. Here, we performed longitudinal field sampling, mNGS, and SURPI analysis of individual *D. occidentalis* ticks collected in Mendocino County, California from 2011–2014 to explore their viral and bacterial diversity and to detect both known and potential tick-borne pathogens afflicting humans.

## Materials and Methods

### Study Area

Ticks were collected at the University of California Hopland Research and Extension Center (HREC) in northwestern California, USA. The HREC is a 5,358-acre, multi-purpose agricultural sciences research facility located on the western slopes of the Mayacamas Mountains in the Russian River valley. The topography consists of rolling hills interspersed with ravines, and ranges in elevation from 152 to 914 m. Grassland, woodland-grass, dense woodland and chaparral comprise more than 95% of the ground cover. Cool, moist winters and hot, dry summers characterize the climate.

The principal vegetational types sampled for presence of ticks were chaparral and adjacent grassland. Buck brush (*Ceanothus cuneatus*), chamise (*Adenostoma fasciculatum*), coyote brush (*Baccharis pilularis*), leather oak (*Quercus durata*), manzanita (*Arctostaphylos* sp.) and toyon (*Heteromeles arbutifolia*) composed the chaparral. Canarygrass (*Phalaris* sp.), quaking grass (*Briza maxima*), great brome (*Bromus diandrus*) and slender wild oat (*Avena barbata*) were the principal grasses.

### Tick Collections

Host-seeking *D. occidentalis* adults were collected by flagging chaparral or grass abutting a dirt road with a 1.0 × 1.0 m^2^ white-flannel cloth attached to a wooden dowel. The linear extent of trailside vegetation sampled each time that yielded ticks was less than 100 m. On each sampling occasion, two study investigators flagged vegetation for one hour apiece in mid-morning on 19 June 2011, 21 May 2013 and 24 May 2014. Adult ticks were identified to species and sex, and then stored at −80 °C until RNA extraction.

### Tick RNA sequencing library prep

Ticks were washed for 15 min in a solution of Wescodyne (Fisher Scientific) followed by 15 min in a solution of 96% ethanol to remove surface contaminants. Excess solution was absorbed and ticks were air-dried prior to manipulation under sterile conditions. Each tick was individually cut in half lengthwise using sterile razor blades; one half of each tick was processed for metagenomic analysis, while the other half was stored frozen at −80 °C for follow-up studies.

Each half-tick (and 1 no-template control) was individually incubated in a 441 µl solution of 30 mAU/mL proteinase K (Qiagen), 5 U/mL chitinase (Sigma-Aldrich), 15 ng/µl carrier RNA (Qiagen), 210 ul buffer AL (Qiagen) and nuclease-free water at 37 C for 20 min, then individually crushed with a separate sterile mini-pestle and 0.1mm silica beads from Lysing matrix B tubes (MP Biomedicals). RNA from supernatant was then extracted using QIAmp viral RNA extraction kit (Qiagen) following the manufacturer’s instructions and eluted in 40ul nuclease-free water. A no-template control consisting of buffer was extracted in parallel to control for cross-contamination. Half of the extract was treated with Turbo DNase (Ambion) for 20 min at 37 °C, inactivated and converted to cDNA using random hexamer primers in a 30 μL reaction with Superscript III reverse transcriptase (Invitrogen). Second-strand synthesis was done using Sequenase (Affymetrix) according to the manufacturer’s instructions. The resulting double-stranded cDNA was then purified using the QIAamp MinElute Kit (Qiagen), and the total eluate was used as input for generating an individually barcoded tick metagenomic sequencing library using Nextera XT (Illumina) following the manufacturer’s instructions.

### Metagenomic sequencing and analysis

Tick sequencing libraries were quantified and pooled according to Qubit (Invitrogen) spectrophotometer readings, and the size and molarity of all library pools assessed by Bioanalyzer (Agilent). Sequencing was performed over 4 lanes of 100 base pair (bp) paired-end run on a HiSeq. 2500 (Illumina).

Metagenomics sequencing datasets were analyzed for pathogens using SURPI, a bioinformatics pipeline for pathogen detection and discovery^6^. We first computationally subtracted tick sequences from the metagenomic data using the nucleotide aligner SNAP^7^. As the *D. occidentalis* genome was not available at the time, whole genome sequences for *Rhipicephalus microplus*, *Ixodes ricinus*, *Ixodes scapularis*, and the mitochondrial genomes of 33 hard- and soft-bodied ticks, including *Dermacentor nitens* and *Dermacentor silvarum* were downloaded from GenBank and used as the tick database for computational subtraction. Viruses, bacteria, fungi, and microparasites were identified by using SNAP to map remaining reads after tick host subtraction to the National Center for Biotechnology Information nucleotide nt reference database (March 2015), and reads with ≥90% identity to microbial reference sequences were identified using the nucleotide aligner SNAP^7^ with an edit distance of 12. Low-stringency translated nucleotide alignments to reference sequences in the GenBank viral protein database (June 2013) was then performed using RAPSearch^8^ to detect novel organisms with divergent genomes on the basis of remote amino acid homology. A total of 93 reads matched to PCR amplicons and/or genomes corresponding to viruses handled or cultured in the laboratory. These viral reads were attributed to contamination, and included sequences from dengue virus, hepatitis C virus, human pegivirus 2, influenza A virus, and enterovirus D68.

### PCR confirmation

PCR confirmation and identification of *Rickettsia* spp. reads down to the species level by amplicon sequencing were performed using a 2x PCR master mix (Thermo Scientific) following manufacturer’s instructions with gltA primers and ompA semi-nested primers. PCR confirmation and speciation of *Anaplasma* spp. reads were attempted following the same protocol and using previously published primers targeting the 16S ribosomal RNA (rRNA) gene^9^.

### *De novo* assembly of viral genomes and phylogenetic analysis

*De novo* assembly of the viral genome was performed from the individual tick sample that contained the highest number of reads mapping to the new bunyavirus. A viral sequence “seed” was chosen for each of the 3 putative bunyaviral segments (S, M, and L), and iterative *de novo* contig assembly using all reads not aligning to tick genomes (“host-subtracted” reads) was performed using the PRICE assembler at default settings^10^. Assembled scaffolds corresponding to each of the 3 segments were then manually examined and curated for gaps and misassemblies using Geneious software v6.0^11^. To assemble viral genomes for each individual tick, host-subtracted reads were mapped to the prototype scaffolds using BLASTn at an e-value cutoff of 10^−8^.

Phylogenetic analysis was performed on all viral proteins from the novel bunyaviruses identified here and corresponding proteins from all *Nairovirus* and *Phlebovirus* species in GenBank. Alignments were generated using 8 iterations of the MUSCLE aligner^12^. Phylogenetic topologies were constructed using a maximum likelihood approach with PhyML software. Substitution models were automatically selected based on the Akaike information criterion using the smart model selection algorithm^13^. A subtree pruning and regrafting method was used to refine topological rearrangements^14^. Branch support was evaluated using a Bayesian-like evaluation of the approximate likelihood ratio test^15^.

### Data availability

Illumina HiSeq sequencing data has been submitted to the NCBI Sequence Read Archive (SRA) under accession numbers SRP093777. PCTN and PCTP genomes have been deposited into NCBI GenBank (accession numbers KU933933–KU933937). The *Francisellaceae* 16s rRNA sequence has been deposited into NCBI GenBank (accession number KX890130).

## Results

### Tick collection and RNA sequencing

**Figure 1:**
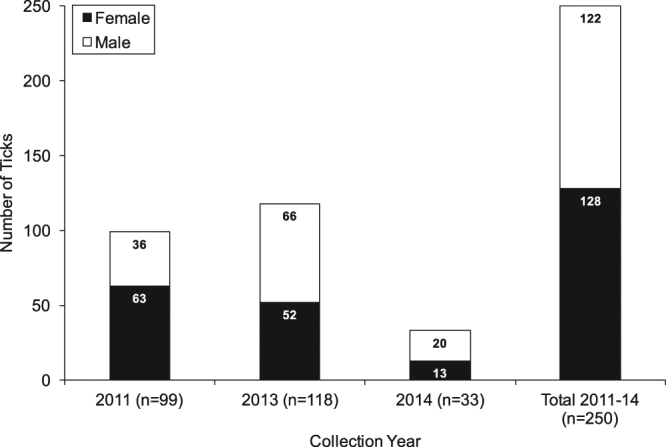
Number of adult *Dermacentor occidentalis* ticks collected by date and sex.

**Figure 2:**
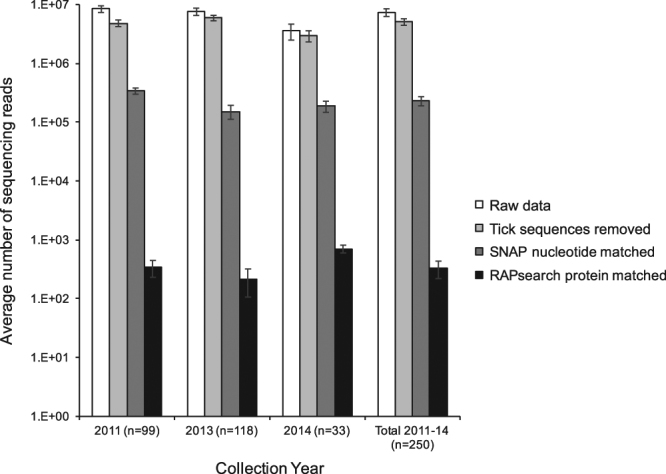
Metagenomic sequencing average read count and alignment to reference nucleotide and protein databases by collection date. The average number of raw data reads (white), reads that do not align to tick genomes (light gray), reads aligning to the complete NT GenBank database by SNAP nucleotide alignment (dark gray), and reads aligning to the GenBank viral database by RAPsearch by protein aligment (black) are presented. Error bars represent the standard error of the mean.

A total of 250 adult *D. occidentalis* ticks were collected, 99 in June 2011, 118 in May 2013, and 33 in May 2014. Overall, 122 ticks were males and 128 were females (Fig. 1). An average of 7.3 (±5.06) million reads per tick were obtained, with an average of 76.2% host (tick) sequences being aligned and subtracted by SURPI, followed by an average of 230,000 reads per sample aligning to a sequence in the NCBI nt database and an average of 327 reads per sample mapping to a viral protein database by translated nucleotide alignment (Fig. 2).

### Identification of two novel *Bunyaviridae*

No known zoonotic viral pathogens were identified by high-stringency nucleotide alignment in any of the 250 ticks. However, translated nucleotide alignment to the GenBank viral protein database revealed the presence of reads aligning to divergent nairoviruses and phleboviruses in 172 ticks (Table 1). The near-complete (>99%) viral genome was recovered from the tick with the highest number of *Nairovirus* (PCTN_N097_2011_M) or *Phlebovirus* (PCTP_105_2014_F) reads. In total, viral reads corresponding to the novel nairovirus and/or phlebovirus were detected in 205 ticks. No reads to PCTN or PCTP were identified in a negative “no-template” control library processed and sequenced in parallel.

**Table 1:** Number of *D. occidentalis* adults testing positive for RNA for human, insect and plant viruses by collection date and sex.

	Collection date	19 June 2011		21 May 2013		24 May 2014		2011–14
	Tick sex	Female (n = 63)	Male (n = 36)	Female (n = 52)	Male (n = 66)	Female (n = 13)	Male (n = 20)	Total (n = 250)
Insect-borne Bunyaviruses	*Nairovirus*	38 (60.3%)	21 (58.3%)	27 (51.9%)	35 (53.0%)	9 (69.2%)	14 (70.0%)	144 (57.6%)
	*Phlebovirus*	55 (87.3%)	35 (97.2%)	9 (17.3%)	14 (21.2%)	8 (61.5%)	14 (70.0%)	135 (54.0%)
Insect viruses	*Alphabaculovirus*	1 (1.6%)	1 (2.8%)	1 (1.9%)	0 (0.0%)	0 (0.0%)	0 (0.0%)	3 (1.2%)
	*Iteradensovirus*	0 (0.0%)	1 (2.8%)	0 (0.0%)	0 (0.0%)	0 (0.0%)	0 (0.0%)	1 (0.4%)
	*Muscavirus*	11 (17.5%)	5 (13.9%)	1 (1.9%)	2 (3.0%)	2 (15.4%)	0 (0.0%)	21 (8.4%)
Insect-borne Plant viruses	*Badnavirus*	0 (0.0%)	0 (0.0%)	0 (0.0%)	1 (1.5%)	0 (0.0%)	0 (0.0%)	1 (0.4%)
	*Caulimovirus*	0 (0.0%)	1 (2.8%)	0 (0.0%)	0 (0.0%)	0 (0.0%)	0 (0.0%)	1 (0.4%)
	*Citrivirus*	0 (0.0%)	0 (0.0%)	0 (0.0%)	1 (1.5%)	1 (7.7%)	0 (0.0%)	2 (0.8%)
	*Potexvirus*	0 (0.0%)	0 (0.0%)	1 (1.9%)	1 (1.5%)	0 (0.0%)	1 (5.0%)	3 (1.2%)
	*Potyvirus*	1 (1.6%)	0 (0.0%)	0 (0.0%)	0 (0.0%)	0 (0.0%)	0 (0.0%)	1 (0.4%)
	*Tobamovirus*	0 (0.0%)	2 (5.6%)	0 (0.0%)	0 (0.0%)	0 (0.0%)	0 (0.0%)	2 (0.8%)

### Nairovirus

#### Genome

**Figure 3:**
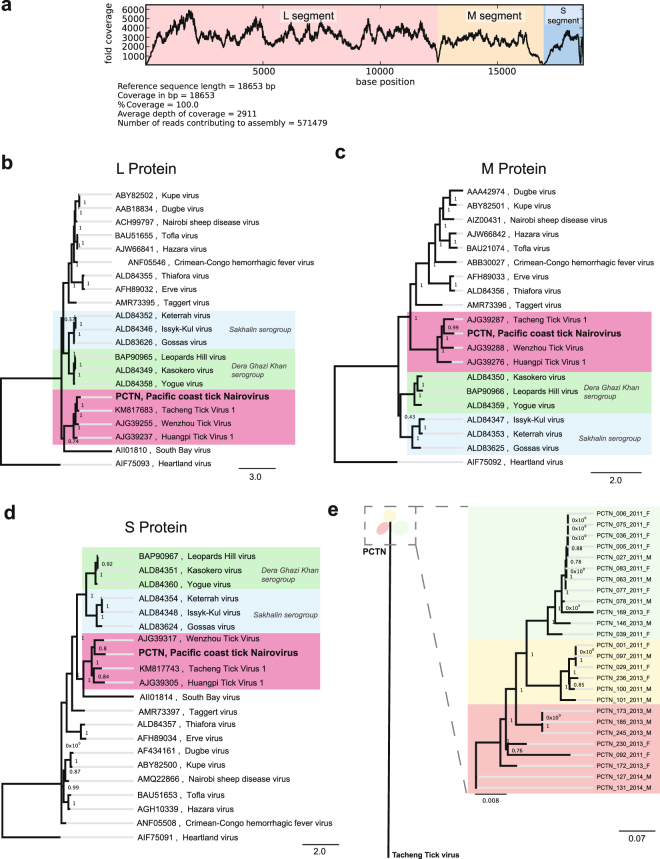
Genomic analysis of the PCT *Nairovirus*, (**a**) Genome size, composition and coverage isolated from a single tick. (**b**–**d**) Phylogeny of PCT *Nairovirus* complete proteins compared to other *Nairovirus* species. (**e**) Phylogeny of 26 PCT *Nairovirus* genomes isolated from single tick.

The 3 segments of a novel *Nairovirus*, provisionally named Pacific Coast tick nairovirus (PCTN), were identified by translated nucleotide analysis. Isolate PCTN_097_2011_M had the highest number of reads to this new viral genome, and thus was chosen as the prototype (GenBank accession numbers: KU933933, KU933934, and KU933935). The near complete segments L, M and S were 12,461 base pairs (bp), 4,509 bp, and 1,739 bp in length, respectively (Fig. 3a). The RNA-dependent RNA polymerase (RdRP), the glycoprotein, and the nucleocapsid protein comprised 4,061 amino acids (aa), 1,360 aa, and 493 aa, respectively. The typical 5′ terminal sequences of *Nairovirus* (AGAGTTTGT) and *Phlebovirus* (ACACAAG)^16^ were not detected by deep sequencing analysis, likely because the Nextera transposase library preparation method used here (Illumina) only generates sequences >50 bp from each distal end of a fragment.

#### Prevalence

**Figure 4:**
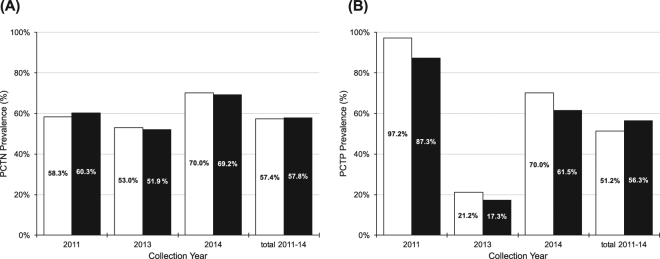
Proportion of male (white) and female (black) ticks positive for PCTN (**A**) and PCTP (**B**) in 2011, 2013, and 2014.

Reads to PCTN were detected in 144 (57.6%) ticks, but not in the no-template control library, indicating a low likelihood of cross-contamination. A tight range of prevalence occurred across multiple years, with 51.9 to 70% of male and female ticks infected at any sampling time point (Table 1, Fig. 4). Differences in annual prevalence or sex distribution were not significant using Pearson’s chi-square test (*p* = 0.19 and 0.94, respectively).

#### Phylogeny

Bayesian phylogeny was performed on each of the PCTN protein sequences against 18 reference *Nairovirus* (with the exception of the phylogeny for the glycoprotein for which only 17 reference *Nairovirus* sequences were available), using corresponding sequences from a *Phlebovirus* (Heartland virus) as an outgroup. We found that PCTN clustered in a novel monophyletic clade formed by tick viruses recently identified in China, closer to representatives from the Dera Ghazi Khan and Sakhalin serogroups than to South Bay virus, which has been recently identified in ticks from North America (Fig. 3b,c and d). PCTN genomes with over 95% coverage were recovered from 26 ticks. PCTN genome diversity was low in comparison to its closest *Nairovirus* species relative, Tacheng Tick virus 1 (Fig. 3e). Average pairwise nucleotide identity between PCTN and Tacheng tick virus 1 was 53.6% (within the range of nucleotide identities reported between *Nairovirus* species of 32.3–62.5%^17^), yet pairwise identity between any 2 PCTN genome isolates was ≥92.7% (Supplementary Table 1). PCTN diversity could be separated into 3 distinct clusters, independent of collection date and the sex of the tick.

### Phlebovirus

#### Genome

**Figure 5:**
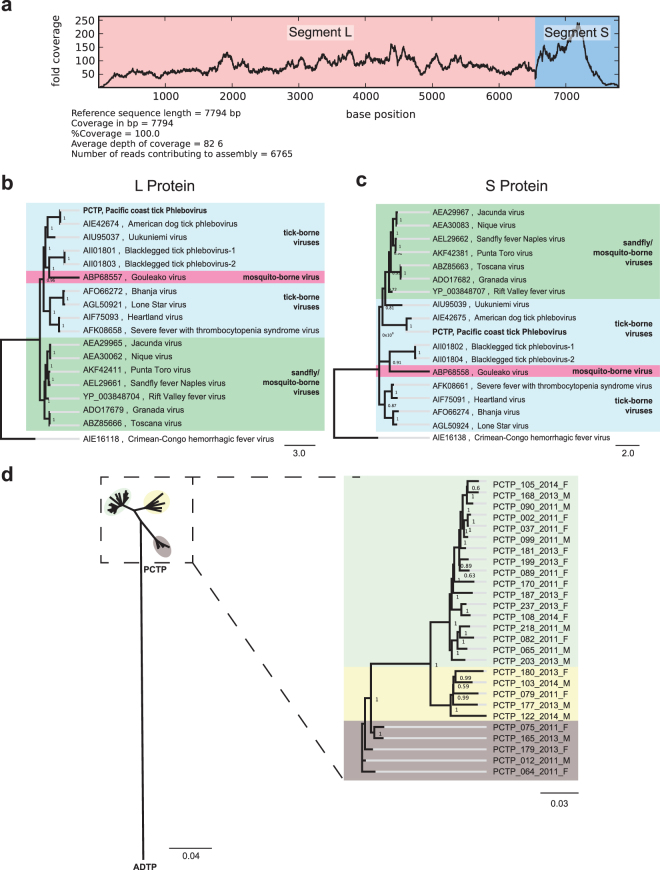
Genomic analysis of PCT *Phlebovirus*, (**a**) Genome size, composition and coverage isolated from a single tick. (**b**–**d**) Phylogeny of PCT *Phlebovirus* complete proteins compared to 15 reference *Phlebovirus* spp. (**e**) Phylogeny of 26 PCT *Phlebovirus* genomes isolated from single ticks.

Two segments of a novel *Phlebovirus*, provisionally named Pacific Coast Tick phlebovirus (PCTP), were identified by translated nucleotide analysis. Isolate PCTP_105_2014_F had the highest number of reads to this new virus and was chosen as the prototype PCTP (GenBank accession numbers: KU933936, and KU933937). The near complete segments L and S were 6,553 bp and 1,538b p in length, respectively (Fig. 5a). The RNA-dependent RNA polymerase (RdRP) and the nucleocapsid protein contained 2,104 aa and 357 aa, respectively. No sequences related to a *Phlebovirus* glycoprotein were found.

#### Prevalence

Reads to PCTP were detected in 135 of 250 ticks (54%), but not in the no-template control library, with females collected in 2011 yielding the highest prevalence (35 of 36, 97.2%) and females collected in 2013 the lowest (9 of 52, 17.3%) (Table 1 and Fig. 4B). Using Pearson’s chi-square test, prevalence differed significantly across collection years (*p* < 0.00001), but not between sexes (*p* = 0.46).

#### Phylogeny

Bayesian phylogeny was performed on each of the PCTP protein sequences against 16 representative phleboviruses (Fig. 5), and all 76 described species of *Phlebovirus* (Supplementary Figs 1 and 2), using sequences from a *Nairovirus* (Crimean-Congo hemorrhagic fever virus, CCHFV) as the outgroup. We found that PCTP clustered with other tick-borne phleboviruses, with American Dog Tick virus as its closest relative. Phylogenetic analyses of both the RdRP and the nucleocapsid proteins revealed identical tree topologies (Fig. 5b and c). PCTP genomes were recovered from 27 ticks with over 95% coverage. PCTP genomic diversity was small in comparison to its closest relative in the genus *Phlebovirus*, American Dog Tick virus (Fig. 5d). The pairwise identity between any 2 PCTP genome isolates was found to ≥86.8% (Supplementary Table 2), whereas the 68.8% nucleotide identity between PCTP and American dog tick phlebovirus was within the range of nucleotide identities between other *Phlebovirus* species (50.4–80.3%)^18^. PCTP diversity could be separated into 3 distinct clusters, and was not associated with collection date or tick sex.

### Identification of invertebrate and insect-borne plant viruses

Reads with >90% identity at the nucleotide level matched known invertebrate and insect-borne viruses (Table 1). Three invertebrate virus genera were identified. One tick had reads to the genus *Iteradensovirus*, 2 ticks had reads to the genus *Alphabaculovirus*, and 21 ticks had reads to the genus *Muscavirus*. Ten ticks were positive for reads to one of 6 plant viral genera: *Badnavirus, Caulimovirus, Citrivirus, Potexvirus, Potyvirus* and *Tobamovirus*.

### Identification of tick-borne bacteria

**Figure 6:**
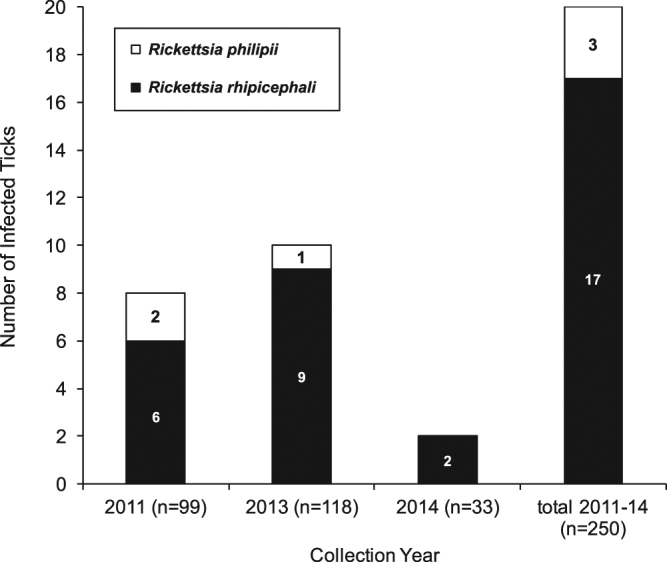
Number of ticks positive for 2 *Rickettsia* species by collection date. The number of ticks positive for *R. rhipicephali* (black) and *R. philipii* (white) is given.

For this analysis, we considered only families of tick-borne bacteria known to contain species pathogenic for mammals, and for which matching reads were not detected in the negative control. These included matches to the genera *Francisella* and *Rickettsia* using SURPI (Table 2). *Francisella* reads were identified in 174 of 250 ticks (69.6%). Assembly of a *Francisellaceae* 16 s rRNA sequence (GenBank accession number KX890130) from isolate Docc2013-187-M resulted in a 1,479 bp fragment with 99.5–100% identity to the previously sequenced *Francisella* endosymbiont of *D. occidentalis* and <98.6% identity to nonsymbiotic *Francisella* species (Supplementary Table 3). Nineteen ticks were positive for *Rickettsia* spp., and PCR followed by amplicon Sanger sampling identified 16 ticks as corresponding to *R. rhipicephali* and 3 ticks to *R. philipii* (Fig. 6). Two ticks were positive for *Anaplasma* spp., but 16 s rRNA PCR confirmation for speciation was negative and the identified reads were to other non-coding regions.

**Table 2:** Number of ticks testing positive for RNA from tick-borne bacteria by collection date and sex.

Collection date	June 19, 2011		May 21, 2013		May 24, 2014		2011–14
Tick sex	Female (n = 63)	Male (n = 36)	Female (n = 52)	Male (n = 66)	Female (n = 13)	Male (n = 20)	Total (n = 250)
*Francisella*	58 (92.1%)	34 (94.4%)	22 (42.3%)	33 (50.0%)	11 (84.6%)	16 (80.0%)	174 (69.6%)
*Rickettsia*	6 (9.5%)	2 (5.6%)	2 (3.8%)	7 (10.1%)	1 (7.7%)	1 (5.0%)	19 (7.6%)

## Discussion

Many tick-associated human illnesses remain undiagnosed^19^ and the prevalence, diversity, and pathogenicity of novel tick-borne agents is poorly understood^20^, underscoring the need for ongoing microbial surveillance in tick vectors. Here we used metagenomic next-generation sequencing (mNGS) to individually screen 250 *D. occidentalis* adults collected in northern California from 2011–2014 for the presence of viruses and bacteria. The genomes corresponding to 2 novel viruses belonging to the genera *Nairovirus* and *Phlebovirus* in the family *Bunyaviridae* were recovered, and sequences mapping to one or both of these novel viruses were detected in 20–91% of sampled ticks. We also detected using mNGS several bacteria previously shown to circulate in the tick vector, including non-pathogenic endosymbionts in the *Francisella* and *Rickettsia* genera and *R. philipii*, a pathogen associated with eschar-associated febrile illness in humans.

*D. occidentalis* is one of the most geographically widespread human-biting ixodid ticks in California. Indeed, it reportedly occurs in 54 of the 58 counties in the state^21^, and is also present in Oregon and northern Baja California, Mexico. The adult ticks frequently infest humans, deer, horses and cattle, whereas the larvae and nymphs parasitize many species of rodents and lagomorphs (hares, pikas, and rabbits). *D. occidentalis* is a likely vector of Colorado tick fever virus and the bacterial agents of human granulocytic anaplasmosis (HGA), Q-fever, Rocky Mountain spotted fever (RMSF), and tularemia; more recently, this tick was identified as the primary vector of *R. philipii* ^22^.

PCTN is phylogenetically closest to Tacheng Tick virus I, a recently discovered virus isolated from *Dermacentor marginatus* ticks in Tacheng, China^23^. PCTP is most similar to American Dog Tick phlebovirus, a virus sequenced from *Dermacentor variabilis* ticks from New York State, USA in 2014^16^. It is not known if any of these viruses (PCTN, PCTP, Tacheng Tick virus I or American Dog Tick virus) are pathogenic to humans.

Notably, PCTN was detected in 57.6% (95% CI [56.6%, 58.6%]) and PCTP in 54% (95%CI [49.9%, 58.1%]) of the *D.occidentalis* ticks tested in this study. The observed prevalences of PCTN and PCTP are much higher than those corresponding to tick-borne viruses known to be pathogenic to humans. For example, among the *Bunyaviridae*, CCHFV infection prevalences in *Hyalomma* ticks were 4.9 to 5.6% in Bulgaria and Iran^24,25^, and the prevalence of Heartland virus in adult *Amblyomma americanum* was 0.11% in Missouri, USA^26^. Among the *Flaviviridae*, tick-borne encephalitis virus mean infection prevalences in adult *Ixodes ricinus* ticks were 0.55–4.48% in Northern Europe, and Powassan virus infection prevalences in *Ixodes scapularis* were 0–4.2% in Connecticut, USA^27^. Such striking disparities in tick-infection prevalences between established human pathogens and our novel viral species suggest that PCTN and PCTP might be either commensals or endosymbionts of *D. occidentalis*. Although little is known about viral symbionts in ticks, bacterial endosymbionts are exceedingly common in ticks, if not ubiquitous^28–30^. Reports of insect-specific arboviruses with high prevalence have only recently emerged. Badu virus, a Phlebovirus, was amplified from 39–100% of mosquito pools^31^. Culex Flavivirus was amplified from 145/210 (69%) of mosquito pools^32^. These 2 novel arboviruses have been reported to be insect-specific^31,32^, as they could not be amplified in vertebrate cell lines. Further investigation including viral cell culture and serological surveillance studies of PCTN and PCTP will be needed to determine whether these viruses are capable of infecting vertebrates.

By multiple sequence alignment, the respective average pairwise genome identity between strains in individual ticks was 97% and 91.9% for PCTN and PCTP, respectively.

The high 97% sequence similarity in PCTN contrasts with the reported diversity of geographically disparate mammalian genomes of CCHFV, another *Nairovirus*, ranging from 20–31%^33^. However, analysis of 89 CCHFV strains from human infections localized in Kosovo revealed only 0–1.5% nucleotide difference over a partial S segment sequence^34^, as compared to 0.4–7.2% for PCTN over the same fragment. Thus, it appears that local evolutionary changes may be limited for CCHFV and PCTN, but that there may be wider geographic and temporal diversity, as is the case for CCHFV. The average pairwise identity of PCTP isolates is more similar to that of Toscana virus (TOSV, 91.7%) than to RVFV (97.7%)^35^, although both viruses are known human pathogens^36^. It has been hypothesized that TOSV, in contrast to RVFV, does not complete its replication cycle in mammalian hosts, and thus is not subject to the higher purifying selection pressure associated with RVFV^35^. Similar to TOSV, the higher diversity of PCTP relative to RVFV implies an absence of an amplifier vertebrate host.

None of the 9 viral genera infecting arthropods or plants detected in this study are known to be transmitted by ticks, and not enough sequencing reads were available to reconstruct complete viral genomes. These reads were likely not reagent contaminants as they were not detected in the negative no-template control; however, it is possible that some of the reads correspond to environmental contaminants carried internally by ticks. Indeed, ticks spend a lot of time in direct contact with vegetation during their host-seeking activity periods.

Earlier studies of *D. occidentalis* ticks in the same geographic area demonstrated that *A. phagocytophilum*, the agent of HGA, was detected in only one (0.2%) of 513 ticks from 2003-10, whereas *A. bovis* and *A. ovis* were detected in 8 (5.1%) and one (0.64%) of 156 ticks in 2005^1^, respectively. In the current study, no unambiguous reads to *Anaplasma* spp. were detected.

RNA sequences homologous to the genus *Francisella* were identified in 174 (69.6%) of the ticks. *Francisella* reads from our RNAseq data most closely matched the genome of *F. persica*, with 94% identity overall. *F. persica*, earlier described as *Wolbachia persica*, was the first tick endosymbiont ever identified and isolated. However, 16 s rRNA sequence alignment revealed even higher similarity to the known *Francisella* endosymbiont of *D. occidentalis*. Unfortunately, the genome for this *Francisella* species has not yet been sequenced, although based on 16 S rRNA homology, it is closely related to the symbiont from 3 other *Dermacentor* ticks, i.e., *D. andersoni*, *D. hunteri*, and *D. variabilis* ^37^.

*Rickettsia* reads were identified in 19 (7.6%) of the ticks. Most of those ticks carried *R. rhipicephali* (17 of 250, 6.8%), which is moderately pathogenic for guinea pigs^38^ but is not known to cause human illness. On the other hand, three (1.2%) ticks were positive for *R. philipii*, a spotted fever group rickettsia causing an illness characterized by eschar, fever, headache and regional lymphadenopathy^22^. Two other studies previously looked at *Rickettsia* prevalence in *D. occidentalis* ticks collected at the University of California Hopland Research and Extension Center (HREC) in northwestern California, USA^22,39^. In 1981, microimmunofluorescence serotyping of cultured *Rickettsia* isolates from *D. occidentalis* determined that 51 and 2 of 233 (21.9% and 0.86%) tested positive for *R. rhipicephali* and *R. philipii*, respectively^39^. Quantitative PCR and sequencing of the *ompA* gene determined that 18 and 1 of 89 *D. occidentalis* nymphs (20.2% and 1.12%) tested positive for *R. rhipicephali* and *R. philipii*, respectively^22^. Thus, prevalence results for *R. philipii* at the same location during a 30-year interval are strikingly similar, even though different detection tools were used. In the present study, two ticks were positive for *R. philipii* in 2011, one in 2013, and none in 2014, but year-to-year variation were not significant (Fisher’s exact test, 2-tailed test, *p* = 0.73). In southern California*, R. philipii* prevalence in *D. occidentalis* was found to be 6 to 7.7% by molecular typing^40,41^. These regional differences in prevalence may reflect dissimilar testing procedures, the number of ticks assayed, year-to-year or habitat variation in biotic and abiotic factors that drive tick ecology and pathogen transmission dynamics.

We conclude that metagenomic sequencing of individual adult *D. occidentalis* ticks is a powerful tool for determining the presence and prevalence of novel viruses and known bacteria at the molecular level, including the tick-borne pathogen *R. philippi*. Metagenomics is rapidly revealing the extent of viral genomic diversity in arthropods, with 1,445 RNA viruses discovered in 2016^42^. A number of these new viruses were found in the class Arachnida (comprising ticks) and belonged to the Bunya-Arena clade and the *Hepe-Virga* clade, which includes the viral genera *Nairovirus*, *Phlebovirus*, *Citrivirus*, *Potexvirus*, and *Tobamovirus* identified in this study. The tick virome is more genetically diverse than currently described, and longitudinal metagenomic surveillance of viral ecology in ticks warrants further investigation. Follow-up studies are also needed to determine whether the newly discovered PCTN and PCTP viruses infect vertebrate hosts or if they harbor zoonotic potential.

## Electronic supplementary material

Supplementary Information
