# Supplementary material for: Metagenomic-based Surveillance of Pacific Coast tick Dermacentor occidentalis Identifies Two Novel Bunyaviruses and an Emerging Human Ricksettsial Pathogen
Source: Sci Rep. 2017 Sep 25;7:12234. doi: 10.1038/s41598-017-12047-6 (PMC5612965; doi:10.1038/s41598-017-12047-6)
Supplement: Supplementary file 1 — Supplementary Information [file 41598_2017_12047_MOESM1_ESM.pdf]

SUPPLEMENTARY INFORMATION

Metagenomic-based Surveillance of the Pacific Coast tick *Dermacentor occidentalis* Identifies Two Novel Bunyaviruses and an Emerging Human Rickettsial Pathogen

Jerome Bouquet[1], Michael Melgar[1], Andrea Swei[3], Eric Delwart[4], Robert S Lane[2] and Charles Y Chiu[1,5,6*]

[1]Department of Laboratory Medicine, University of California, San Francisco, CA 94143, USA

[2]Department of Environmental Science, Policy and Management, University of California, Berkeley, CA 94720, USA

[3]Department of Biology, San Francisco State University, San Francisco, CA 94132, USA

[4]Blood Systems Research Institute, San Francisco, CA 94118, USA

[5]UCSF-Abbott Viral Diagnostics and Discovery Center and Department of Medicine, University of California, San Francisco, CA, USA

[6]Department of Medicine, Division of Infectious Diseases, University of California, San Francisco, CA 94107

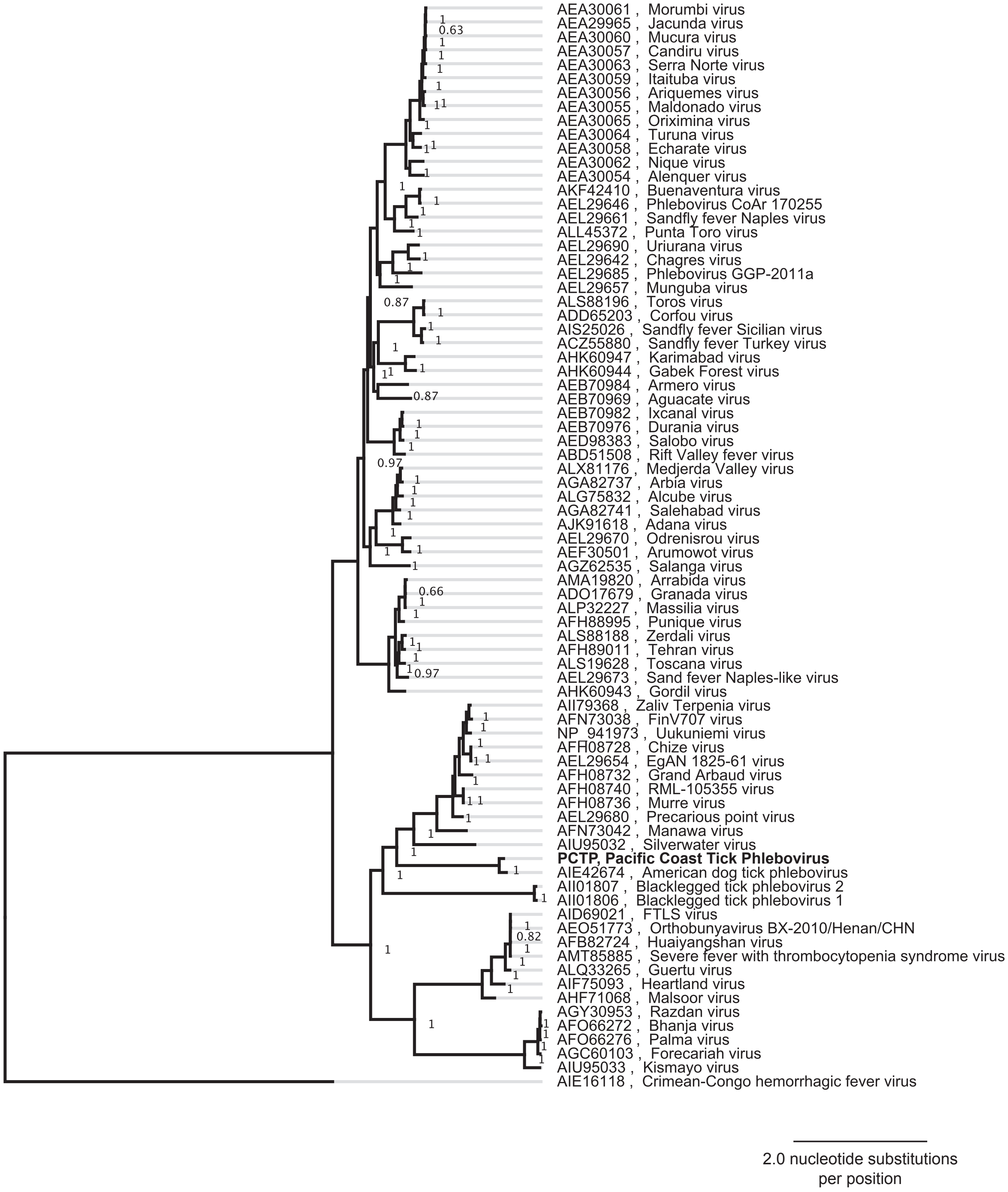

Supplementary Figure 1. Phylogeny of the L (large) protein of PTCP (Pacific Tick Coast phlebovius) compared to all 76 sequenced phleboviruses in NCBI GenBank.

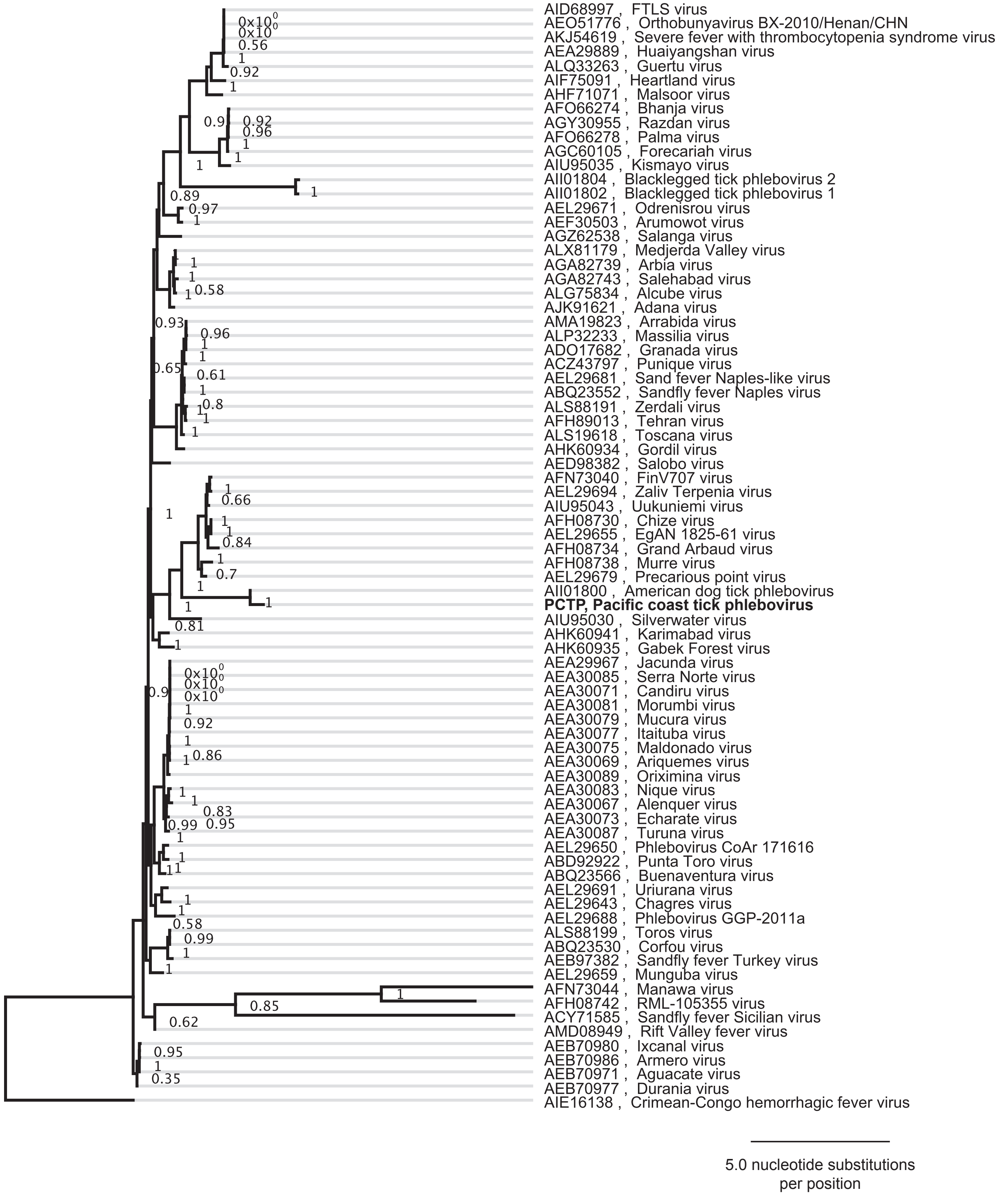

Supplementary Figure 2. Phylogeny of the N (nucleocapsid) protein of PTCP (Pacific Tick Coast phlebovirus) compared to all 76 sequenced phleboviruses in NCBI GenBank.

Supplementary Table 1 – Percent identity matrix from the alignment of 26 near complete concatenated genomes (95%) of PCTN virus with Tacheng tick virus 1.

	Docc2013-169-F	Docc2013-146-M	Docc2011-039-F	Docc2011-005-F	Docc2011-078-M	Docc2011-006-F	Docc2011-036-F	Docc2011-027-M	Docc2011-077-F	Docc2011-075-F	Docc2011-063-M	Docc2011-083-F	Docc2011-101-M	Docc2013-236-F	Docc2011-100-M	Docc2011-029-F	Docc2011-097-M	Docc2011-001-F	Docc2011-092-F	Docc2013-230-F	Docc2014-127-M	Docc2014-131-M	Docc2013-172-F	Docc2013-173-M	Docc2013-185-M	Docc2013-245-M	Tacheng Tick virus 1
Docc2013-169-F		93.8	94.3	94.9	95.1	94.9	95	95	95.1	95	95	95	93.4	93.4	93.4	93.5	93.5	93.6	92.7	93.3	93.4	93.6	93.6	93.8	93.4	93.6	52.4
Docc2013-146-M	93.8		97.6	97.8	98	97.8	97.8	97.8	98	97.9	97.9	97.8	96.4	95.6	96.2	96	96	96	95.3	96.1	96.2	96.5	96.5	96.5	96.2	96	53.6
Docc2011-039-F	94.3	97.6		98.2	98.5	98.3	98.4	98.4	98.6	98.5	98.5	98.4	97.3	96.5	97	96.9	96.9	96.8	96.2	96.6	97	97.4	97.3	97.3	96.7	96.6	53.8
Docc2011-005-F	94.9	97.8	98.2		99	99	99.1	99.1	99.2	99.1	99.1	99.1	97.1	96.5	96.8	96.7	96.6	96.6	95.9	96.6	96.6	97.2	97	97.2	96.8	96.7	53.7
Docc2011-078-M	95.1	98	98.5	99		99	99.1	99.2	99.4	99.3	99.3	99.3	97.4	96.6	97.1	97	97	96.9	96.3	96.7	97.1	97.5	97.4	97.4	96.8	96.7	53.8
Docc2011-006-F	94.9	97.8	98.3	99	99		99.2	99.1	99.2	99.1	99.2	99.1	97.2	96.4	96.9	96.7	96.8	96.7	96	96.5	96.9	97.2	97.2	97.2	96.7	96.6	53.8
Docc2011-036-F	95	97.8	98.4	99.1	99.1	99.2		99.3	99.4	99.4	99.4	99.3	97.3	96.6	97.1	96.9	97	96.9	96.2	96.7	97.1	97.5	97.4	97.4	96.9	96.8	53.8
Docc2011-027-M	95	97.8	98.4	99.1	99.2	99.1	99.3		99.5	99.4	99.3	99.4	97.4	96.6	97.2	96.9	97	96.9	96.3	96.7	97	97.6	97.4	97.4	96.8	96.7	53.9
Docc2011-077-F	95.1	98	98.6	99.2	99.4	99.2	99.4	99.5		99.5	99.6	99.6	97.5	96.7	97.1	97	97.1	96.9	96.3	96.8	97.1	97.6	97.5	97.5	96.8	96.7	53.9
Docc2011-075-F	95	97.9	98.5	99.1	99.3	99.1	99.4	99.4	99.5		99.5	99.5	97.3	96.6	97	96.9	96.9	96.8	96.1	96.6	96.9	97.5	97.3	97.4	96.8	96.7	53.8
Docc2011-063-M	95	97.9	98.5	99.1	99.3	99.2	99.4	99.3	99.6	99.5		99.5	97.4	96.7	97.2	97	97.1	97	96.3	96.7	97.1	97.5	97.4	97.4	96.9	96.8	53.8
Docc2011-083-F	95	97.8	98.4	99.1	99.3	99.1	99.3	99.4	99.6	99.5	99.5		97.4	96.6	97.1	96.9	97	96.9	96.2	96.7	97	97.4	97.4	97.4	96.8	96.7	53.8
Docc2011-101-M	93.4	96.4	97.3	97.1	97.4	97.2	97.3	97.4	97.5	97.3	97.4	97.4		97.9	97.7	97.7	97.1	97.4	94.8	96.3	96.6	97.3	97	97.3	96.7	96.5	53.3
Docc2013-236-F	93.4	95.6	96.5	96.5	96.6	96.4	96.6	96.6	96.7	96.6	96.7	96.6	97.9		98.3	98.4	98.5	98.4	95.5	96.1	96	96.5	96.4	96.9	96.5	96.4	53.7
Docc2011-100-M	93.4	96.2	97	96.8	97.1	96.9	97.1	97.2	97.1	97	97.2	97.1	97.7	98.3		98	97.6	97.7	94.8	96.2	96.4	97.1	96.8	97.3	96.7	96.5	53.2
Docc2011-029-F	93.5	96	96.9	96.7	97	96.7	96.9	96.9	97	96.9	97	96.9	97.7	98.4	98		98.3	98.4	95	96.2	96.4	96.9	96.8	97.2	96.6	96.4	53.4
Docc2011-097-M	93.5	96	96.9	96.6	97	96.8	97	97	97.1	96.9	97.1	97	97.1	98.5	97.6	98.3		98.2	94.8	96.1	96.2	97	96.7	97.2	96.6	96.5	53.5
Docc2011-001-F	93.6	96	96.8	96.6	96.9	96.7	96.9	96.9	96.9	96.8	97	96.9	97.4	98.4	97.7	98.4	98.2		94.8	96.1	96.3	96.9	96.7	97.2	96.6	96.5	53.4
Docc2011-092-F	92.7	95.3	96.2	95.9	96.3	96	96.2	96.3	96.3	96.1	96.3	96.2	94.8	95.5	94.8	95	94.8	94.8		96.6	96.7	97.2	97.1	96.7	96.1	96	53.2
Docc2013-230-F	93.3	96.1	96.6	96.6	96.7	96.5	96.7	96.7	96.8	96.6	96.7	96.7	96.3	96.1	96.2	96.2	96.1	96.1	96.6		97.2	97.6	97.7	97.6	97.5	97.3	53.6
Docc2014-127-M	93.4	96.2	97	96.6	97.1	96.9	97.1	97	97.1	96.9	97.1	97	96.6	96	96.4	96.4	96.2	96.3	96.7	97.2		99.3	98.1	97.5	97	96.9	53.6
Docc2014-131-M	93.6	96.5	97.4	97.2	97.5	97.2	97.5	97.6	97.6	97.5	97.5	97.4	97.3	96.5	97.1	96.9	97	96.9	97.2	97.6	99.3		98.6	97.9	97.3	97.2	54
Docc2013-172-F	93.6	96.5	97.3	97	97.4	97.2	97.4	97.4	97.5	97.3	97.4	97.4	97	96.4	96.8	96.8	96.7	96.7	97.1	97.7	98.1	98.6		98	97.4	97.3	53.7
Docc2013-173-M	93.8	96.5	97.3	97.2	97.4	97.2	97.4	97.4	97.5	97.4	97.4	97.4	97.3	96.9	97.3	97.2	97.2	97.2	96.7	97.6	97.5	97.9	98		99	98.8	53.8
Docc2013-185-M	93.4	96.2	96.7	96.8	96.8	96.7	96.9	96.8	96.8	96.8	96.9	96.8	96.7	96.5	96.7	96.6	96.6	96.6	96.1	97.5	97	97.3	97.4	99		98.9	53.7
Docc2013-245-M	93.6	96	96.6	96.7	96.7	96.6	96.8	96.7	96.7	96.7	96.8	96.7	96.5	96.4	96.5	96.4	96.5	96.5	96	97.3	96.9	97.2	97.3	98.8	98.9		53.6
Tacheng Tick virus 1	52.4	53.6	53.8	53.7	53.8	53.8	53.8	53.9	53.9	53.8	53.8	53.8	53.3	53.7	53.2	53.4	53.5	53.4	53.2	53.6	53.6	54	53.7	53.8	53.7	53.6	

Supplementary Table 2 – Percent identity matrix from the alignment of 27 near complete concatenated genomes (95%) of PCTP virus with American dog tick phlebovirus.

	UCTP_218_2011_M	UCTP_170_2011_F	UCTP_075_2011_F	UCTP_012_2011_M	UCTP_105_2014_F	UCTP_181_2013_F	UCTP_199_2013_F	UCTP_002_2011_F	UCTP_187_2013_F	UCTP_180_2013_F	UCTP_079_2011_F	UCTP_168_2013_M	UCTP_099_2011_M	UCTP_065_2011_M	UCTP_089_2011_F	UCTP_122_2014_M	UCTP_082_2011_F	UCTP_037_2011_F	UCTP_103_2014_M	UCTP_179_2013_F	UCTP_203_2013_M	UCTP_090_2011_M	UCTP_177_2013_M	UCTP_064_2011_F	UCTP_237_2013_F	UCTP_165_2013_M	UCTP_108_2014_F	American dog tick phlebovirus
UCTP_218_2011_M		95	94.7	92.8	94	86.8	87.6	87.5	87.9	87.8	87.5	87.9	88.4	88.7	88.1	90.1	88.6	88.8	89.7	88.7	89.4	90	90.4	91	90.8	90.7	91.3	68.4
UCTP_170_2011_F	95		95.3	93.7	94.9	87.8	88.7	88.3	89	88.9	88.5	88.8	89.5	89.3	89.1	91.6	89.3	89.4	90.4	89.4	90.4	90.7	90.7	91.5	91.6	91.5	91.9	68.9
UCTP_075_2011_F	94.7	95.3		94.9	95	88.2	89.5	89.3	89.4	89.4	89.5	89.8	90.1	90.1	89.6	91.6	90.3	90.3	91.1	90.2	90.9	91.9	92	92.4	92.4	92.2	92.7	69.4
UCTP_012_2011_M	92.8	93.7	94.9		94.6	87.6	88	88	87.3	88.4	89	89.4	89.4	88.3	88.4	90.2	88.4	89	89.3	89.2	89.5	91.2	89.9	90.1	90.1	90.4	90.4	68.9
UCTP_105_2014_F	94	94.9	95	94.6		88.2	88.3	88.5	88	88.6	89.3	89.7	89.6	88.8	88.5	90.6	89.1	89.3	90	89.6	89.7	91.5	90.4	90.3	90.6	91.1	90.7	69.2
UCTP_181_2013_F	86.8	87.8	88.2	87.6	88.2		90.2	90.4	88.1	89	90.8	89.4	90.1	88.9	88.9	92.4	88.5	88.9	90	89.9	89.4	91.2	89.9	90.5	90.4	91	90.6	67.8
UCTP_199_2013_F	87.6	88.7	89.5	88	88.3	90.2		90.8	88	89.7	91.7	89.9	90.4	88.7	89.7	93	89.2	89.9	90.1	89.6	90.6	91.6	91	91.4	91	91.2	90.9	67.5
UCTP_002_2011_F	87.5	88.3	89.3	88	88.5	90.4	90.8		88.4	89.8	91.6	90.1	90.9	88.9	89.5	93.2	88.9	89.8	91.2	91.5	90.4	92.2	90.9	91.7	91.4	91.4	91	67.3
UCTP_187_2013_F	87.9	89	89.4	87.3	88	88.1	88	88.4		91.1	88.6	91.2	92.2	91.5	91.5	91.2	91.8	91.7	92.5	91.5	92.7	93.2	93.2	94	93.9	93.6	93.7	68
UCTP_180_2013_F	87.8	88.9	89.4	88.4	88.6	89	89.7	89.8	91.1		90.7	93	93.8	92	92.7	91.7	91.6	92.4	92.8	92.7	93.2	94.6	93.3	94.1	93.5	94	93.9	67.9
UCTP_079_2011_F	87.5	88.5	89.5	89	89.3	90.8	91.7	91.6	88.6	90.7		91.7	91.6	89.7	90	93.4	89.6	90.6	90.5	90.7	90.9	92.9	91	91.6	91.3	91.5	91.5	68.4
UCTP_168_2013_M	87.9	88.8	89.8	89.4	89.7	89.4	89.9	90.1	91.2	93	91.7		94.1	92.3	92.4	91.5	92.2	93.1	93	93.1	93.5	95.5	93.5	95	93.8	94.2	94.3	68.6
UCTP_099_2011_M	88.4	89.5	90.1	89.4	89.6	90.1	90.4	90.9	92.2	93.8	91.6	94.1		93	93.1	92.5	92.4	93.5	93.8	93.8	94.2	95.4	94.3	95	94.7	95.1	95	68.9
UCTP_065_2011_M	88.7	89.3	90.1	88.3	88.8	88.9	88.7	88.9	91.5	92	89.7	92.3	93		92.1	91.6	92.9	93.4	94.2	92.4	94.2	94	95	94.8	95	95.2	95.5	67.8
UCTP_089_2011_F	88.1	89.1	89.6	88.4	88.5	88.9	89.7	89.5	91.5	92.7	90	92.4	93.1	92.1		92.1	91.7	92	92.6	93.2	93.4	95	93.4	94.2	93.7	94.2	94.2	68.3
UCTP_122_2014_M	90.1	91.6	91.6	90.2	90.6	92.4	93	93.2	91.2	91.7	93.4	91.5	92.5	91.6	92.1		91.9	92.3	92.7	92.2	93.2	93.6	93.5	94.5	94.2	94	94.1	69.2
UCTP_082_2011_F	88.6	89.3	90.3	88.4	89.1	88.5	89.2	88.9	91.8	91.6	89.6	92.2	92.4	92.9	91.7	91.9		93.2	93.8	92.1	94	94.2	94.8	95	95.1	95.2	95.6	68.3
UCTP_037_2011_F	88.8	89.4	90.3	89	89.3	88.9	89.9	89.8	91.7	92.4	90.6	93.1	93.5	93.4	92	92.3	93.2		94.4	92.7	94.8	94.9	95.4	94.1	95.5	94.9	95.4	68.9
UCTP_103_2014_M	89.7	90.4	91.1	89.3	90	90	90.1	91.2	92.5	92.8	90.5	93	93.8	94.2	92.6	92.7	93.8	94.4		94.5	94.6	95.2	95.9	95.6	96.4	96.5	97.1	68.9
UCTP_179_2013_F	88.7	89.4	90.2	89.2	89.6	89.9	89.6	91.5	91.5	92.7	90.7	93.1	93.8	92.4	93.2	92.2	92.1	92.7	94.5		93.7	96.3	94.3	94.9	94.4	94.8	94.4	68.7
UCTP_203_2013_M	89.4	90.4	90.9	89.5	89.7	89.4	90.6	90.4	92.7	93.2	90.9	93.5	94.2	94.2	93.4	93.2	94	94.8	94.6	93.7		95.6	95.9	95.9	96.3	96	96.7	69.1
UCTP_090_2011_M	90	90.7	91.9	91.2	91.5	91.2	91.6	92.2	93.2	94.6	92.9	95.5	95.4	94	95	93.6	94.2	94.9	95.2	96.3	95.6		95.8	96.4	96	96.2	96.4	70.3
UCTP_177_2013_M	90.4	90.7	92	89.9	90.4	89.9	91	90.9	93.2	93.3	91	93.5	94.3	95	93.4	93.5	94.8	95.4	95.9	94.3	95.9	95.8		96.5	97.4	97.1	97.5	69.4
UCTP_064_2011_F	91	91.5	92.4	90.1	90.3	90.5	91.4	91.7	94	94.1	91.6	95	95	94.8	94.2	94.5	95	94.1	95.6	94.9	95.9	96.4	96.5		97.2	97.3	97	69.8
UCTP_237_2013_F	90.8	91.6	92.4	90.1	90.6	90.4	91	91.4	93.9	93.5	91.3	93.8	94.7	95	93.7	94.2	95.1	95.5	96.4	94.4	96.3	96	97.4	97.2		97.3	97.8	69.3
UCTP_165_2013_M	90.7	91.5	92.2	90.4	91.1	91	91.2	91.4	93.6	94	91.5	94.2	95.1	95.2	94.2	94	95.2	94.9	96.5	94.8	96	96.2	97.1	97.3	97.3		97.7	69.6
UCTP_108_2014_F	91.3	91.9	92.7	90.4	90.7	90.6	90.9	91	93.7	93.9	91.5	94.3	95	95.5	94.2	94.1	95.6	95.4	97.1	94.4	96.7	96.4	97.5	97	97.8	97.7		69.6
American dog tick ph	68.4	68.9	69.4	68.9	69.2	67.8	67.5	67.3	68	67.9	68.4	68.6	68.9	67.8	68.3	69.2	68.3	68.9	68.9	68.7	69.1	70.3	69.4	69.8	69.3	69.6	69.6	

Supplementary Table 3 – Percent identity matrix from the alignment of the 16s rRNA sequence of *Francisella* endosymbiont of *Dermacentor occidentalis* to those of 31 other *Francisella* species.

	-	JQ031629	AY375402	AY805304	FJ468434	AY805307	AY375399	AY375401	AY375403	AY375395	EU332820	AY375407	KT382876	JQ942365	JQ740890	NR_116944	CP009353	KC170748	KC170749	KC170755	KC170751	KC170750	CP013022	KJ522773	KC170771	KP659194	CP006875	CP009436	CP009574	JF290376	EF685351	CP010427
**Docc2013-187-F_Franciseallaceae_16s**		**100**	**100**	**100**	**99.6**	**98.6**	**99.2**	**99.4**	**99.5**	**99.3**	**98.7**	**98.7**	**98.9**	**98.6**	**98.5**	**97.8**	**97.6**	**98.5**	**98.5**	**98.3**	**98.2**	**98**	**98.6**	**98.4**	**98.2**	**98.1**	**96.2**	**96.5**	**96.7**	**96.1**	**97.1**	**93.2**
Francisella endosymbiont of Dermacentor occidentalis isolate G021209MR-4	**100**		100	100	99.1	99.6	99.1	99.1	99.6	98.9	99.1	99.2	99.1	98.9	98.7	99.3	99.3	98.2	98	97.8	97.8	97.5	99.1	98.4	98.4	98.4	98.2	98.4	98.7	98.2	98.2	95.1
Francisella endosymbiont of Dermacentor occidentalis clone DoC-2.E16s	**100**	100		100	99.6	99.3	99.2	99.4	99.5	99.3	98.7	98.7	98.9	98.6	98.5	97.6	97.3	98.5	98.4	98.2	98.1	97.9	98.3	98.4	98.2	98.2	95.6	96	96.2	95.7	96.9	93.4
Francisella endosymbiont of Dermacentor occidentalis strain 2031093	**100**	100	100		99.6	99.7	99.3	99.5	99.6	99.3	98.7	98.7	98.9	98.6	98.5	98.6	98.2	98.5	98.5	98.3	98.2	98	98.4	98.4	98.2	98.1	96.4	96.8	96.5	96	96.8	93.9
Francisella endosymbiont of Dermacentor andersoni	**99.6**	99.1	99.6	99.6		99.7	99.6	99.8	99.6	99.4	98.9	98.7	98.9	98.6	98.5	98.5	98.2	98.4	98.3	98.1	98	97.8	98.4	98.4	98.2	98.1	96.4	96.8	96.5	95.9	96.7	93.8
Francisella endosymbiont of Dermacentor variabilis	**98.6**	99.6	99.3	99.7	99.7		99.1	99.3	99.7	99	98.8	98.6	99	98.7	98.5	96.9	96.6	98.4	98.3	98.1	98.1	97.9	97.6	98.6	98.2	98.2	95.1	95.4	95.5	95.5	96.5	92.1
Francisella endosymbiont of Dermacentor hunteri	**99.2**	99.1	99.2	99.3	99.6	99.1		99.5	99.2	99.1	98.5	98.5	98.5	98.2	98.1	97.4	97.1	97.9	97.9	97.7	97.6	97.4	98.1	98	97.9	97.8	95.4	95.8	95.9	95.4	96.5	93.1
Francisella endosymbiont of Dermacentor nitens	**99.4**	99.1	99.4	99.5	99.8	99.3	99.5		99.4	99.3	98.7	98.7	98.7	98.5	98.3	97.6	97.3	98.2	98.1	97.9	97.8	97.7	98.3	98.2	98.1	98	95.6	96	96.1	95.6	96.7	93.3
Francisella endosymbiont of Dermacentor occidentalis clone 02-241.E16s	**99.5**	99.6	99.5	99.6	99.6	99.7	99.2	99.4		99.2	98.7	98.9	98.9	98.6	98.5	97.7	97.3	98.3	98.2	98	97.9	97.8	98.3	98.4	98.2	98.2	95.7	96.1	96.2	95.7	96.8	93.6
Francisella endosymbiont of Dermacentor albipictus	**99.3**	98.9	99.3	99.3	99.4	99	99.1	99.3	99.2		99.1	98.4	98.5	98.2	98.1	97.3	97	97.9	97.9	97.7	97.8	97.4	98	98	97.9	97.8	95.5	95.7	96.1	95.5	96.5	93.1
Francisella-like endosymbiont of Rhipicephalus microplus	**98.7**	99.1	98.7	98.7	98.9	98.8	98.5	98.7	98.7	99.1		97.7	98	97.9	97.7	97.9	97.5	97.4	97.3	97.1	97.2	96.8	97.6	97.6	97.5	97.5	96.1	96.4	96.2	95.7	96.3	93.5
Francisella endosymbiont of Amblyomma maculatum	**98.7**	99.2	98.7	98.7	98.7	98.6	98.5	98.7	98.9	98.4	97.7		98.7	98.4	98.3	97.6	97.2	98.1	98.2	98	97.9	97.8	98.2	98.2	98.2	98.2	95.6	96	96.3	95.9	96.7	93.5
Francisella endosymbiont of Amblyomma geoemydae	**98.9**	99.1	98.9	98.9	98.9	99	98.5	98.7	98.9	98.5	98	98.7		99.5	99.3	99	98.6	98.9	98.6	98.5	98.4	98.2	98.8	98.6	98.7	98.7	96.6	97.1	96.8	96.3	97	94.2
Francisella-like endosymbiont of Dermacentor reticulatus	**98.6**	98.9	98.6	98.6	98.6	98.7	98.2	98.5	98.6	98.2	97.9	98.4	99.5		99.8	99.1	98.7	98.7	98.4	98.2	98.1	97.9	98.7	98.2	98.4	98.4	96.7	97.1	96.8	96.3	97.1	94.4
Francisella-like endosymbiont of Ixodes ricinus	**98.5**	98.7	98.5	98.5	98.5	98.5	98.1	98.3	98.5	98.1	97.7	98.3	99.3	99.8		98.9	98.6	98.6	98.3	98.1	98	97.8	98.6	98.1	98.3	98.3	96.5	96.9	96.6	96.1	96.9	94.3
Francisella hispaniensis	**97.8**	99.3	97.6	98.6	98.5	96.9	97.4	97.6	97.7	97.3	97.9	97.6	99	99.1	98.9		99.7	98.5	98.4	98.2	98.1	97.9	98.2	98.3	98.3	98.5	97.7	98.4	96.6	95.9	97.9	94.8
Francisella tularensis	**97.6**	99.3	97.3	98.2	98.2	96.6	97.1	97.3	97.3	97	97.5	97.2	98.6	98.7	98.6	99.7		98.1	98	97.8	97.7	97.5	98.1	97.9	98.1	98.3	97.8	98.4	96.8	96	97.9	94.9
Francisella endosymbiont of Dermacentor atrosignatus	**98.5**	98.2	98.5	98.5	98.4	98.4	97.9	98.2	98.3	97.9	97.4	98.1	98.9	98.7	98.6	98.5	98.1		99.5	99.4	99.3	99.1	98.4	98.2	98	97.9	96.4	96.8	96.5	95.9	96.7	93.7
Francisella endosymbiont of Dermacentor auratus isolate	**98.5**	98	98.4	98.5	98.3	98.3	97.9	98.1	98.2	97.9	97.3	98.2	98.6	98.4	98.3	98.4	98	99.5		99.8	99.7	99.5	98.3	97.9	97.7	97.6	96.3	96.7	96.4	95.8	96.6	93.8
Francisella endosymbiont of Haemaphysalis shimoga	**98.3**	97.8	98.2	98.3	98.1	98.1	97.7	97.9	98	97.7	97.1	98	98.5	98.2	98.1	98.2	97.8	99.4	99.8		99.5	99.4	98.1	97.8	97.5	97.4	96.1	96.5	96.2	95.6	96.5	93.6
Francisella endosymbiont of Rhipicephalus haemaphysaloides	**98.2**	97.8	98.1	98.2	98	98.1	97.6	97.8	97.9	97.8	97.2	97.9	98.4	98.1	98	98.1	97.7	99.3	99.7	99.5		99.3	98	97.7	97.5	97.3	96	96.5	96.1	95.5	96.4	93.6
Francisella endosymbiont of Dermacentor compactus	**98**	97.5	97.9	98	97.8	97.9	97.4	97.7	97.8	97.4	96.8	97.8	98.2	97.9	97.8	97.9	97.5	99.1	99.5	99.4	99.3		97.8	97.5	97.3	97.2	95.8	96.3	95.9	95.4	96.2	93.4
Francisella persica	**98.6**	99.1	98.3	98.4	98.4	97.6	98.1	98.3	98.3	98	97.6	98.2	98.8	98.7	98.6	98.2	98.1	98.4	98.3	98.1	98	97.8		97.9	98.1	98	96.8	97.2	97.2	96.6	97.7	94.2
Francisella-like endosymbiont of Hyalomma rufipes	**98.4**	98.4	98.4	98.4	98.4	98.6	98	98.2	98.4	98	97.6	98.2	98.6	98.2	98.1	98.3	97.9	98.2	97.9	97.8	97.7	97.5	97.9		97.9	97.9	96.1	96.5	96.2	95.7	96.6	94
Francisella endosymbiont of Amblyomma varanense	**98.2**	98.4	98.2	98.2	98.2	98.2	97.9	98.1	98.2	97.9	97.5	98.2	98.7	98.4	98.3	98.3	98.1	98	97.7	97.5	97.5	97.3	98.1	97.9		99.7	95.9	96.3	96.4	95.8	96.5	93.8
Francisella-like endosymbiont of Rhipicephalus sanguineus	**98.1**	98.4	98.2	98.1	98.1	98.2	97.8	98	98.2	97.8	97.5	98.2	98.7	98.4	98.3	98.5	98.3	97.9	97.6	97.4	97.3	97.2	98	97.9	99.7		96.1	96.4	96.6	96.1	96.7	93.9
Francisella noatunensis	**96.2**	98.2	95.6	96.4	96.4	95.1	95.4	95.6	95.7	95.5	96.1	95.6	96.6	96.7	96.5	97.7	97.8	96.4	96.3	96.1	96	95.8	96.8	96.1	95.9	96.1		98.9	97.8	96.9	99.1	94.8
Francisella philomiragia	**96.5**	98.4	96	96.8	96.8	95.4	95.8	96	96.1	95.7	96.4	96	97.1	97.1	96.9	98.4	98.4	96.8	96.7	96.5	96.5	96.3	97.2	96.5	96.3	96.4	98.9		97.8	97.1	99.1	95.1
Francisella endociliophora	**96.7**	98.7	96.2	96.5	96.5	95.5	95.9	96.1	96.2	96.1	96.2	96.3	96.8	96.8	96.6	96.6	96.8	96.5	96.4	96.2	96.1	95.9	97.2	96.2	96.4	96.6	97.8	97.8		98.9	99.1	93.9
Francisella halioticida strain	**96.1**	98.2	95.7	96	95.9	95.5	95.4	95.6	95.7	95.5	95.7	95.9	96.3	96.3	96.1	95.9	96	95.9	95.8	95.6	95.5	95.4	96.6	95.7	95.8	96.1	96.9	97.1	98.9		98.2	93.6
Francisella piscicida	**97.1**	98.2	96.9	96.8	96.7	96.5	96.5	96.7	96.8	96.5	96.3	96.7	97	97.1	96.9	97.9	97.9	96.7	96.6	96.5	96.4	96.2	97.7	96.6	96.5	96.7	99.1	99.1	99.1	98.2		94.8
Francisella guangzhouensis	**93.2**	95.1	93.4	93.9	93.8	92.1	93.1	93.3	93.6	93.1	93.5	93.5	94.2	94.4	94.3	94.8	94.9	93.7	93.8	93.6	93.6	93.4	94.2	94	93.8	93.9	94.8	95.1	93.9	93.6	94.8	
